# A cultural evolutionary theory that explains both gradual and punctuated change

**DOI:** 10.1098/rsif.2022.0570

**Published:** 2022-11-16

**Authors:** Blai Vidiella, Simon Carrignon, R. Alexander Bentley, Michael J. O’Brien, Sergi Valverde

**Affiliations:** ^1^ Evolution of Networks Lab, Institute of Evolutionary Biology (UPF-CSIC), Passeig Marítim de la Barceloneta 37, 08003 Barcelona, Spain; ^2^ McDonald Institute for Archaeological Research, Downing Street, Cambridge CB2 3ER, UK; ^3^ Department of Anthropology, University of Tennessee, Knoxville, TN 37996, USA; ^4^ Department of Communication, History, and Philosophy and Department of Life Sciences, Texas A&M University–San Antonio, Texas 78224, USA; ^5^ Department of Anthropology, University of Missouri-Columbia, Missouri 65201, USA; ^6^ European Centre for Living Technology (ECLT), Ca’ Bottacin, 3911 Dorsoduro Calle Crosera, 30123 Venezia, Italy

**Keywords:** cultural evolution, punctuated evolution, social learning, transparency, popularity bias

## Abstract

Cumulative cultural evolution (CCE) occurs among humans who may be presented with many similar options from which to choose, as well as many social influences and diverse environments. It is unknown what general principles underlie the wide range of CCE dynamics and whether they can all be explained by the same unified paradigm. Here, we present a scalable evolutionary model of discrete choice with social learning, based on a few behavioural science assumptions. This paradigm connects the degree of transparency in social learning to the human tendency to imitate others. Computer simulations and quantitative analysis show the interaction of three primary factors—information transparency, popularity bias and population size—drives the pace of CCE. The model predicts a stable rate of evolutionary change for modest degrees of popularity bias. As popularity bias grows, the transition from gradual to punctuated change occurs, with maladaptive subpopulations arising on their own. When the popularity bias gets too severe, CCE stops. This provides a consistent framework for explaining the rich and complex adaptive dynamics taking place in the real world, such as modern digital media.

## 1. Introduction

Cumulative cultural evolution (CCE) [1–6], in which innovations accumulate over time through social learning, has been integral to human evolution [7–9] and inter-generational cultural adaptations of small traditional societies [10–15]. When expertise and/or performance are transparent, the rate of CCE correlates with the number of interacting individuals [16–19], in the complexity of forager assemblages [20–22] and in controlled social psychology experiments [23–27] in which small groups can outperform the most skilled/knowledgeable group member on short-term tasks [28–32].

It is not clear what implications CCE studies in small groups should have for larger populations, such as the urban environments humans have lived in for millennia. Extrapolating the hypothesized correlation between CCE and population size, it would seem that larger social learning networks would surface the best technologies [16,33,34], productive organizations [35], government institutions [36,37] and technical knowledge [38–40]. As innovation rate scales with population density [18,41], however, the number of similar options can increase by orders of magnitude, and social learners need to update more frequently to keep up. While copying recent success is an adaptive strategy in a highly variable environment [26,42–44] at some point this capacity may be overwhelmed. When intrinsic pay-offs are no longer transparent, copying recent *popularity* can become a substitute for copying recent success—an understandable shortcut by human psychology evolved for a few hundred social relationships [9,45–48]. Long-run persistence of old information may limit the adoption of adaptations in this scenario [49].

Previous models must be expanded in order to disentangle the interplay between individual and collective levels. Game theory models of binary options, which are effective at characterizing social conformity in animals and small-scale human societies [44,50], cannot adequately reflect modern environments in which numerous ‘games’ are being played concurrently among thousands, if not millions, of agents competing for popularity of their views. The bewildering array of choices is not reducible to a single binary decision, and the transparency of information and social learning criteria vary widely, ranging from zero to a vast range [51,52].

In order to bridge this ‘population gap’ in CCE theory, here we propose a non-equilibrium model that can accommodate any number of similar options as well as varying degrees of information transparency and degree of popularity bias in a population. To recreate a wide range of cultural behaviours, we aim here for the simplest model that relies on the fewest assumptions and parameters. When simulated through heterogeneous interactions, the transparency–popularity link produces a spectrum of collective dynamics spanning from gradual [53] to punctuated change [17,34,54,55] that is essentially independent of specific system properties. Here, discontinuous events do not require highly skilled individuals making ‘great leaps’ [17]. Instead, a frequency-dependent balance of transparency and popularity bias limits the pace of cultural evolution, resulting in stasis associated with spontaneously arising ‘barriers’ that only infrequent events may overcome. In the future, this framework might provide a broad foundation for CCE, allowing it to be adjusted to specific real-world contexts.

## 2. Evolutionary model of discrete choice with social learning

**Figure 1.**
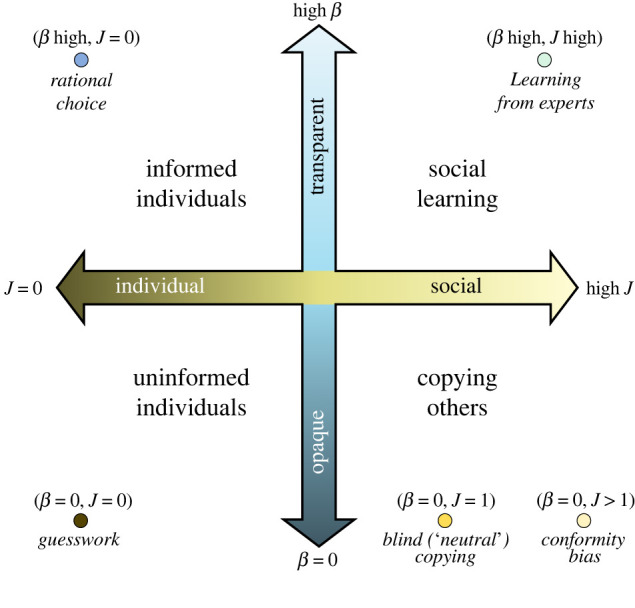
Conceptual ‘dimensions’ of information transmission, with points indicating established models in the continuous space.

Our conceptual model [56] consists of, firstly, the continuum between individual and social learning (horizontal axis in figure 1), and secondly, transparency of information or learning criteria (vertical axis). This model, grounded in discrete theory under social influence [57], unifies a range of approaches, from those emphasizing intrinsic pay-offs of the choices [58], learning from experts or successful individuals [22,42,59], all the way to models that assume copying is done with zero transparency of pay-offs or expertise [52,60–66].

This continuous parameter space includes certain well-known reference points. Rational decisions, per the standard social science model, are individual and transparent (upper left in figure 1), whereas learning from experts, per much of cultural evolutionary theory, is transparent and social (upper right). The bottom half of the continuous space includes random copying (lower right) and guesswork as opaque individual learning (lower left). As these disparate models are reference points within a continuous space of possibility (figure 1), the framework could serve as a conceptual bridge from CCE in small-scale experiments and traditional societies to rapid change under massive, globalized communication.

Although there are many strategies for social learning [59], here we start pragmatically with popularity bias as the horizontal dimension, which we parametrize as *J* [56,67,68]. High *J* means doing as others do. The second key parameter, the vertical dimension, is transparency of learning, which ranges from informed to uninformed [56,69–71]. We parametrize information transparency as *β*, where high *β* enables selection for the best option. As the parameters are continuous, unlimited intermediate scenarios are possible.

Transparency represents an individual’s sensitivity to differences in choice, acting on the intrinsic utility difference between options. Effectively the weight of individual learning, transparency amounts to the extent to which one’s behavioural choice is influenced by the objective pay-off related to that behaviour. In the absence of popularity bias (*J* = 0), the larger the transparency is, the smaller is the variance in decision-making across the alternatives. When transparency is near zero in the absence of popularity bias, choice is random over the choice set, and each option is chosen with the same probability. When transparency is large, the relative values of pay-offs of each choice are high, such that the choice with the highest pay-off is reliably identified.

In each time step of the model, a new set of *N* choices are made. This could represent a new ‘generation’ of *N* agents that replace the previous generation, or it could represent the *N* agents making a new choice to replace the choice held in the previous time step. As both are mathematically equivalent, the model can represent successive generations over long time periods of cultural evolution, or successive time intervals within a time span as agents make Bayesian updates to their decisions. It could also represent successive samples, of size *N* of a large interconnected population, whose choices are evolving through time. Popular choices have their own ‘inertia’ by virtue of stochastic change under popularity bias and/or the persistence of choices with high intrinsic utility under significant transparency.

The model proceeds in each time interval with each of the *N* agents identifying another agent to potentially copy. First, an option, *i*, is observed with probability *Pr*(*i*, *t* + 1) among all the *k*(*t*) different alternatives in the most recent time interval. Following cultural evolutionary theory [72,73], we set this probability to its frequency, *p*_*i*_(*t*), modified by exponent *J*

2.1 $$Pr(i,t+1)\propto p_{i}{\left(t\right)}^{J},$$

where the parameter *J* can bias this frequency-dependent selection. This popularity bias parameter ranges continuously from *J* = 0 for zero frequency effect, to *J* = 1 for probability in strict proportion to frequency (random copying) to *J* > 1 for conformist bias.

Subsequently, agent evaluates the intrinsic utility, *U*_*i*_ of its chosen option *i* and keeps it with probability, *Pr*(*i*, *t* + 1), which is determined logistically by *βU*_*i*_, where *β* is the transparency of choice [22,57,74–76]

2.2 $$Pr(i,t+1)\propto e^{\beta U_{i}+\psi },$$

where *ψ* represents the random Gaussian error in the pay-off estimation [77,78]. Equation (2.2) is typical in formulations of discrete choice theory or quantal response theory [57,79,80]. For parsimony, we leave aside the matter of different intrinsic preferences [81], which would necessitate an additional error term on utilities *U*_*i*_.

Combining the steps, equations (2.1) and (2.2), yields the probability, *Pr*(*i*, *t* + 1), that the agent selects variant *i* at time *t* + 1

2.3 $$Pr(i,t+1)=\frac{1}{Y_{t}}p_{i}{\left(t\right)}^{J}\;e^{\beta U_{i}+\psi },$$

where *Y*_*t*_ is a normalizing term across all *k*(*t*) variants

2.4 $$Y_{t}=\sum_{\;j=1}^{k}p_{\;j}{\left(t\right)}^{J}\;e^{\beta U_{\;j}+\psi }.$$

Alternatively, we can define the propensity $\Pi (U_{i},p_{i})$ of choosing the trait with utility *U*_*i*_ and popularity *p*_*i*_ as follows:

2.5 $$\Pi (U_{i},p_{i})=Pr(i,t+1)Y_{t}=p_{i}{\left(t\right)}^{J}\;e^{\beta U_{i}}\;e^{\psi }.$$

Equations (2.3)–(2.5) span a decision space. The formulation also informs our basic expectations at certain reference points. In the high-transparency realm without social learning (*J* = 0), we recover (2.2), which is a form of bounded rationality. We will see in our results what happens as *J* is increased from this reference point. At the other end of the spectrum, along the zero-transparency extreme, if *β* = 0 and *J* = 0, there is random selection among the *k*(*t*) alternatives, *Pr*(*i*, *t* + 1) = 1/*k*(*t*) + *ψ*. If *β* = 0 and *J* = 1, then it becomes a random copying, or Yule, model, where *Pr*(*i*, *t* + 1) ∝ *p*_*i*_(*t*)e^*ψ*^, i.e. proportional to approximated frequency.

Lastly, a small fraction of agents, *μ*, invents something new by modifying an existing variant *i*, such that its pay-off becomes *U*_*i*_ + *ε*, where the random perturbation *ε* is drawn from a normal distribution with mean zero. In simulations, we vary *μ* from 0.005 to 0.1, consistent with ranges proposed for human invention [82–85]. With new index *k*(*t*) + 1, this new variant becomes part of the pool from which agents may choose in the next time step. Iterating (2.3) generates different probability distribution functions from the same starting point, under conditions of high transparency *β* or high popularity bias *J*.

## 3. Results

The model is multifarious, with an endless number of alternative outcomes. Here, we examine how the major modes of behaviour are affected by *β* and *J*, using a range of population sizes *N* and low mutation rates in our simulations (more comprehensive findings are reported in the electronic supplementary material throughout a grid of *β*–*J* pairings). We also provide analytical predictions for how the rate of utility gain (CCE) varies not just with population size but also with the *J*/*β* ratio.

**Figure 2.**
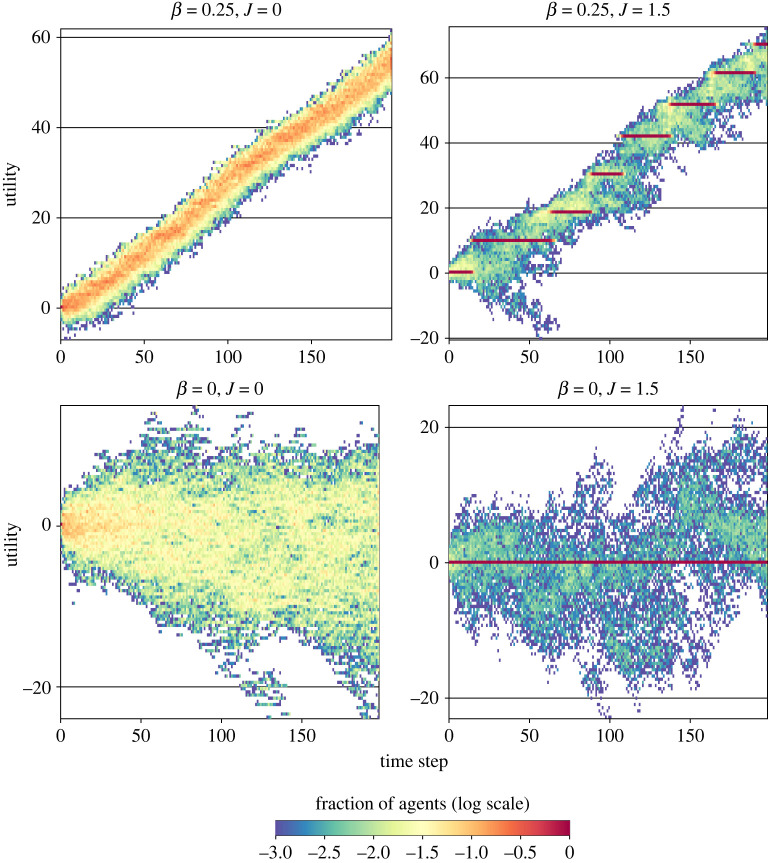
Trait utilities (*y*-axes) through time (*x*-axes) in simulations with different values of transparency (*β*) and popularity bias (*J*). Zero utility is the initial reference value. Colour indicates the relative fraction of agents in logarithmic scale (red = 100%). Note different *y*-axis range for each plot. In each simulation for 200 time steps, *N* = 1000 agents, *μ* = 0.1, *ψ* = 1. Despite the system’s stochastic nature, the reported behaviours are typical (statistically). The electronic supplementary material shows more replicas as well as a strong statistical signature in the rank frequency distributions associated with each behaviour.

Figure 2 shows the evolution of utility values chosen by 1000 agents over 200 time steps, showing a rich diversity of behaviours from this simple model. We identify general evolutionary regimes in the *β*–*J* space, including steady evolution, punctuated evolution, stochastic drift and random noise (clockwise from upper left in figure 2). Under zero transparency (bottom half of figure 2), there is little to no regular increase in utility. Without transparency or popularity bias (*β* = 0, *J* = 0), there is noise (figure 2, lower left). With popularity bias and no transparency (*β* = 0, *J* = 1.5), as in the lower right of figure 2, there are two notable effects. First, a majority of agents never escape their choices (horizontal red bar). Second, among the remaining minority of agents, a drifting ‘consensus’ (greenish band)—a minority of agents overall, but a majority of those not in the red band—is held together by popularity bias. If we were to draw a cross-section at a time step in the *β* = 0, *J* = 1.5 case, the frequency distribution would have multiple modes: a sharp peak at the majority utility value (red bar), but also a wider, secondary mode (greenish band) and other smaller modes, drifting through time.

At the top of figure 2, transparency *β* facilitates a cumulative increase in median utility, as we would expect. For positive transparency (*β* = 0.25) in the upper half of figure 2, the utility values undergo steady increase for *J* = 0 (upper left) and punctuated increases for *J* = 1.5 (upper right). Comparing utility increase by the end of these two simulations under the same value of *β* = 0.25 indicates that CCE can be faster with popularity bias (*J* = 1.5) than without (*J* = 0). The progress at (*β* = 0.25, *J* = 1.5) is not optimal, however—for example, the group drifting into negative utility values in the first 50 time steps and other suboptimal ‘tendrils’ are visible throughout that simulation (figure 2, upper right). This resembles homophily, via sorting into subpopulations around drifting modal utility values, some of which decline. This sorting of the population through popularity bias simply emerges, without having imposed any network or group structure on the model, or any intrinsic preferences instilled among the agents.

**Figure 3.**
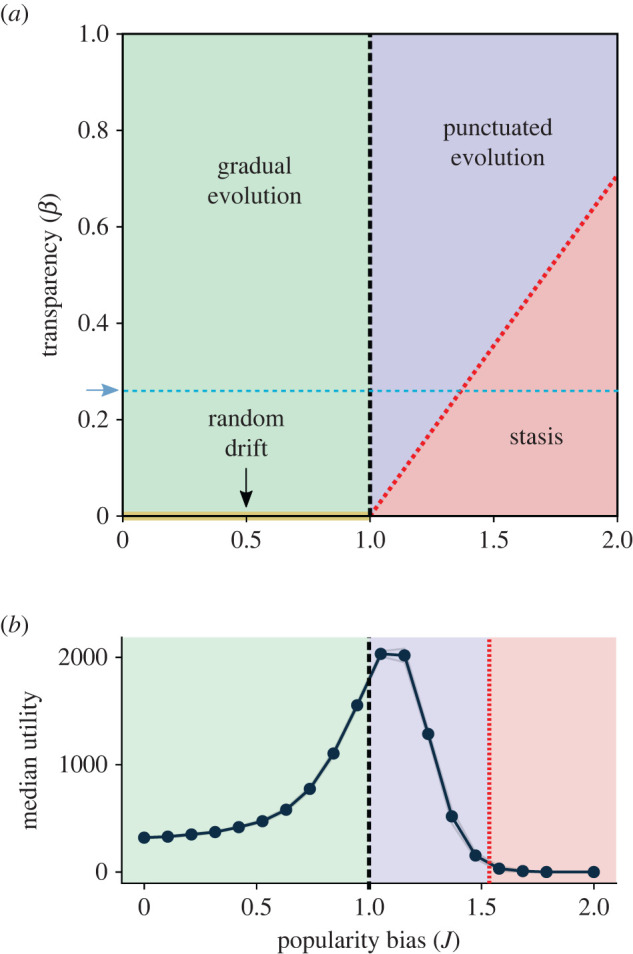
Theoretical boundaries for cumulative cultural evolution defined by equations (3.1) and (5.2). The *J* = 1 transition divides this space between gradual (*J* < 1) and punctuated evolution (*J* > 1). Random drift without selection occurs at *β* = 0 (yellow line), where Δ_*U*__*c*_ ∼ −∞. In the red area in the lower right, there is no CCE as utility barriers are no longer reached. Bottom panel shows average median utility at *β* = 0.25 (blue arrow on top), after *T* time steps in 10 replicates (see Methods). Median utility is maximized at *J* ≈ 1.1 (the so-called ‘soft spot’ of social learning, see text), then declines as *J* is increased further. Errors bars are within the line width.

The optimal rate of CCE should require moderate values of both transparency and popularity bias. Locating the exact optimum *β*−*J* coordinate is not trivial, however, as it depends on the other parameters (*μ*, *N*) and requires a large number of simulations [68]. More coarsely, we can determine that CCE (rate of utility increase) is typically optimized with *J* at or slightly above 1, as long as transparency *β* is positive. Computational simulations show CCE decreasing as *J* is moved away from 1 (figure 3). For large populations and low transparency values, this optimal CCE occurs towards *J* = 1 (discussed below).

**Figure 4.**
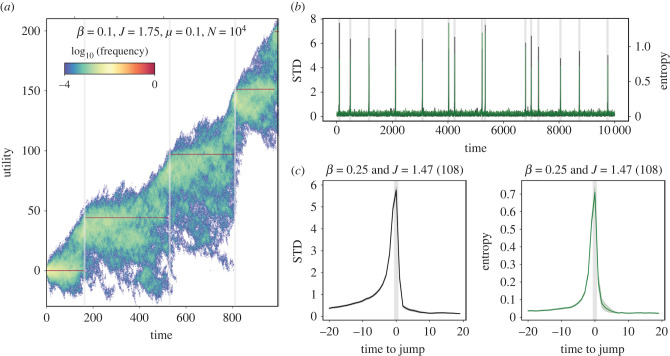
Detecting abrupt jumps in evolutionary cultural dynamics. (*a*) Punctuated pay-off increases take place under low transparency and high conformity. Colour indicates their population frequency (in logarithmic scale, see colour bar). (*b*) Trait diversity peaks very close to sharp jumps in median utility, which take place when the population overcomes the underlying utility barrier (see text). Here, we measure trait diversity using entropy (green curve) and standard deviation (black curve). (*c*) Aggregated statistics collapsing 108 jumps. Simulation parameters: *N* = 10^4^ individuals, *β* = 0.1, *J* = 1.75, *μ* = 0.1, *m* = 1, *ψ* = 1, $T_{\max}=10^{4}\;\text{time steps}$.

Note the increases in utility on the top row of figure 2 are smooth with *J* = 0 and punctuated with *J* = 1.5. In between, we find the transition from gradual to punctuated evolution lies at *J* = 1, which is simple frequency-proportional copying. Increasing *J* above 1, while maintaining sufficient *β* (see below), induces stagnation where plateaus of utility emerge for extended periods, with sporadic jumps to higher plateaus. The population bifurcates upon each new jump, as some agents continue copying the same choice while other agents increase their pay-offs with better (higher utility) choices. We can zoom in to look for early warning signals in time series activity before each jump, such as increased variance before a critical transition [86]. Indeed, for *J* > 1, we do see such signals. In a case with *β* = 0.1 and *J* = 1.75, figure 4*a* shows the utility values of each agent choice over the first 1000 time steps, which increase cumulatively through punctuated leaps. Each red bar indicates a large concentration of individual pay-offs around one utility value; each red point indicates nearly all the agents have copied the same variant (see §3 of the electronic supplementary material for a three-dimensional rendering of the same dataset). Above and below each red plateau in utility are those agents copying variants that differ from the majority. Just before each jump, the variance in utility values, as well as the entropy, increases as a spike (figure 4*b*). Aggregating results from 20 steps before and after each jump, figure 4*c* reveals the abrupt rise and fall of variance and entropy in utility values before and after each jump. This is a recurrent (and statistically repeatable) pattern (figure 4*c*).

**Figure 5.**
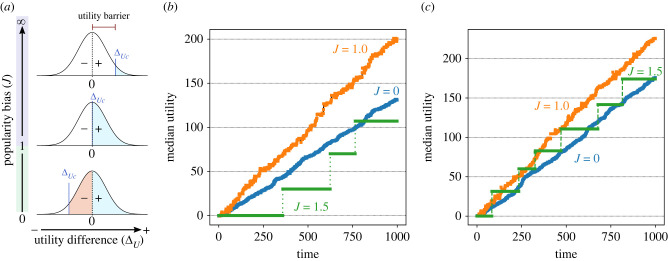
Statistical distribution of innovations and the pace of CCE. (*a*) Gaussian distributions of utility difference $N(\Delta_{U})$ under *J* = 0, *J* = 1 and *J* > 1. Each blue line indicates the utility barrier, Δ_*U*__*c*_. Shading indicates whether random changes are rejected (white), accepted despite negative Δ_*U*_ (red) or accepted with positive Δ_*U*_ (blue). Panels at right show change in median utility during simulations (*ψ* = 0, *N* = 1000, *β* = 0.05) for the three different values of *J* ∈ {0, 1, 1.5} with (*b*) *μ* = 0.05 and (*c*) *μ* = 0.1.

**Figure 6.**
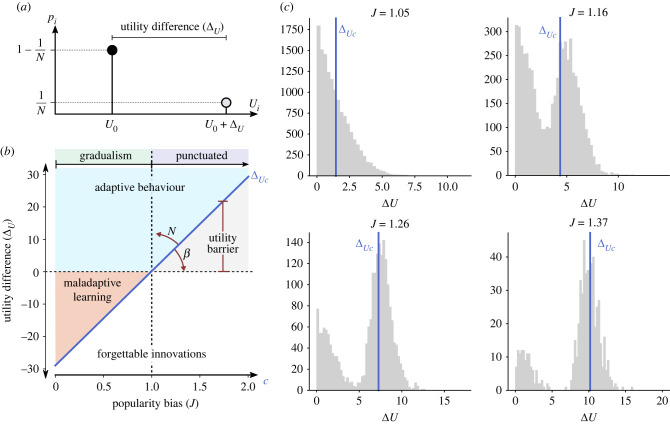
Comparison of theoretical and empirical utility barriers. Our model predicts the emergence of utility thresholds that must be crossed in order for novel variants to be transmitted. (*a*) Utility thresholds (or barriers) can be computed in a basic set-up where the population is centred at utility *U*_0_ (black circle) and there is a single individual at a better variant with utility *U*_0_ + Δ_*U*_ (grey circle). (*b*) Predicted utility difference (Δ_*U*_) versus conformity (*J*). The blue diagonal described by equation (3.1) indicates where adaptations can be maintained (above line) or forgotten (below line). The slope of the diagonal depends on the population size (*N*) and information transparency (*β*). Negative utility barriers are linked with low popularity bias *J* < 1, permitting the fixation of maladaptations; *J* = 1 establishes the boundary between gradual and punctuated change; and positive utility barriers emerge when *J* > 1, limiting the fixation of small adaptations (grey area). (*c*) For various conformity levels, the frequency distribution of the empirical utility barrier is shown (grey bars). Numerical simulations support the theoretical prediction for $\Delta_{U_{c}}$ (solid blue line) (see text). Simulation parameters: *β* = 0.25, *N* = 1000, *T*_max_ = 10000, 10 replicas, *ψ* = 0, *μ* = 0.005.

Another approach to explain the punctuated mode is to consider whether uncommon utility gains are likely to be adopted by others. It has been argued that social systems exist in a condition of stasis for extended periods of time, punctuated by rapid shifts resulting in radical transformations, which are frequently associated with major inventions [34]. Here, we investigate whether punctuated changes are an emergent characteristic of an inherent conflict between transparency and popularity bias. Such ‘utility barriers’ to improvement (figure 5*a*) resemble valleys on a fitness landscape [87], but they are a fundamentally dynamical phenomena caused by cultural evolutionary mechanisms rather than the underlying landscape features. In the mode of punctuated evolution, the population alternates between periods of apparent stasis and abrupt shifts to the next utility *U* + Δ_*U*_ (light grey region in figure 6). When *J* is larger than 1, utility barriers emerge, such that small utility increases below a threshold (Δ_*U*_ < Δ_*U*__*c*_) are not selected. Depending on Δ_*U*__*c*_, the system may be unable to select for new variants of higher utility, due to their low initial frequency (red bar in figure 2, lower right), such that some agents remain stuck with their initial utility value, *U*_0_.

Figure 5 illustrates the effect on CCE of the probability, *P*(*U*_*t*+1_ >*U*_*t*_), that an agent obtains a higher utility in the next time step *t* + 1 (see Methods). For each new invention (produced at rate *μ*), the utility change, (Δ_*U*_), from the agent’s existing variant is drawn from a Gaussian distribution. The Gaussian distribution is centred at zero, so (Δ_*U*_) can be either beneficial (+) or deleterious (−). Figure 5*a* shows three different scenarios of increasing conformity from bottom to top. Below a utility barrier (Δ_*U*__*c*_), the inventions will probably not be copied (figure 5*a*). Increasing popularity bias *J* raises the value of the utility barrier (figure 5*a*, bottom to top), such that popularity bias that is too strong (*J* > 1) will slow down CCE (figure 5*b*,*c*). Conversely, if popularity bias is too weak or absent, CCE is not maximized either (figure 5*b*,*c*). The optimal value is very close to *J* = 1, where maladaptations are forbidden and every possible random change can be selected by the population.

The suggested CCE ‘soft spot’ reflects the utility barrier being minimized at *J* = 1. Overcoming these utility barriers requires the accumulation of sufficient diversity in utility values within the population, as generated by the Gaussian error, *ψ*, in each agent’s pay-off estimation. While population size determines the number of inventions, *μN*, the stronger the popularity bias, *J*, the more trials (inventions) the population needs to make to be likely to overcome the utility barrier Δ_*U*__*c*_ > 0. Since *β* and *J* determine the utility barrier, the relationship between population size and CCE (utility increase rate) will also depend on these parameters. As derived in the Methods section, we can describe the minimal utility improvement, Δ_*U*_, that can reliably be copied by at least one agent in one time step, as

3.1 $$\Delta_{U}\geq {\Delta_{U}}_{c}=\frac{J-1}{\beta }\ln(N-1).$$

Note Δ_*U*__*c*_ = 0 when *J* = 1. As equation (3.1) shows, utility barriers are lowered by transparency *β* and increased by popularity bias *J* and the logarithm of population size *N*.

The larger the popularity bias, the larger transparency *β* needs to be for utility gains to be discovered. Figure 6 further highlights the interplay between these variables, the utility barrier and CCE. In the Δ_*U*_ versus *J* space of figure 6*b*, the prediction of (3.1) is the blue diagonal—changes can occur above this line. Whether changes are positive is delineated by the horizontal at Δ_*U*_ = 0. The space in figure 6*b* is thus divided into four distinct regions: adaptive behaviour (light blue), maladaptive learning (red), forgettable (white) and ‘missed opportunities’ (grey). The adaptive (blue) region yields gradual, steady evolution without significant plateaus. In the red triangular (maladaptive) region, the population may adopt negative utility displacements, which can send subpopulations into decline (see down-sloping tendrils in figure 4*a*). The grey area is called missed opportunities because this is where new inventions with positive utility differences are not adopted because they lie below the utility barrier. These spaces in figure 6*b* change with the ratio Δ_*U*__*c*_ ≈ ln*N*/*β*: as this ratio increases, the boundary rotates counter-clockwise, expanding the region of maladaptive adoptions (red) and missed opportunities (grey), that is, the maladaptive parameter space increases and adaptive space decreases.

When conformity is significant (*J* > 1), utility improvements (Δ_*U*_) below the critical utility barrier Δ_*U*__*c*_, or utility barrier, cannot be widely adopted by the population. The duration of the utility plateaus increases with *J* until eventually, at some conformity limit, *J*_*C*_, the utility barrier is never overcome and CCE ceases. As derived in the Methods (5.2), we can predict how *J*_*C*_ increases with population size, *N*

**Figure 7.**
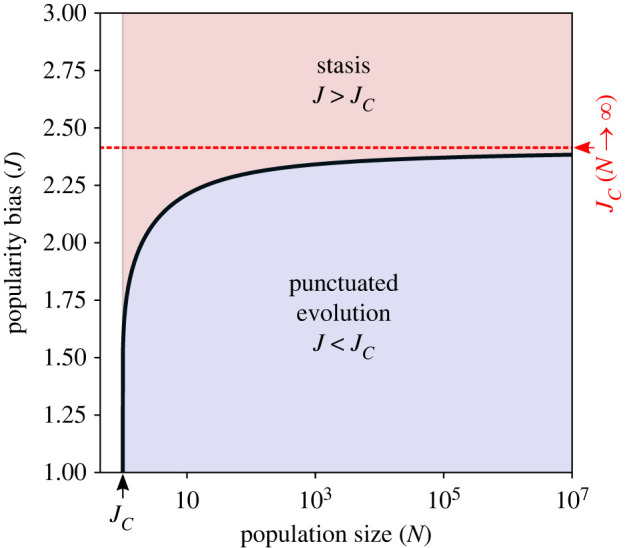
Prediction for the critical value of popularity bias, (*J*_*C*_), separating punctuated evolution from founder effect, using *β* = 1 in (3.2).

3.2 $$J<J_{C}=1+\beta \sqrt{\frac{2\log(N/2)}{\log(N-1)}}$$

which, if we assume that population size is large enough (then *N* − 1 ≈ *N*), indicates that CCE can occur as long as $J\leq 1+\sqrt{2}\beta$. In other words, the crucial ratio is (*J* − 1)/*β*. If this ratio exceeds the variance of the innovation distribution (approx. 1.4 for the Gaussian), CCE ceases. As long as popularity bias is less than *J*_*C*_, CCE can proceed. When the bias exceeds *J*_*C*_, most selections remain stuck at their initial value, *U*_0_. Figure 7 shows how the conformity limit, *J*_*C*_, depends on population size. Increasing population from small to medium sizes pushes the boundary up to higher *J* values, but this effect is asymptotic as the boundary converges to a finite quantity for very large populations.

## 4. Discussion

Unifying distinct aspects of different behavioural disciplines [88], we established a cultural evolutionary model based on the interaction between social learning and transparency of information. As the model is non-deterministic and every simulation is unique, we focus on general effects, their implications for cultural evolution, and future potential modifications. The model is relatively simple, but there are numerous variants, aspects and components of individual and social learning that were left out; we will go through some of them below. This parsimony was essential in order to examine model behaviours, which were already quite rich with just the few parameters used.

Our study demonstrates multiple phases in the space of CCE, ranging from stasis to stochastic drift, gradual change and punctuated change. By integrating these behaviours through few parameters, the model suggests fundamental insights. Firstly, it begins to untangle the much-discussed dependence of CCE on population size [20,21,24,69]. In the model, this relationship depends on a interaction between information transparency *β* and popularity bias *J*. Secondly, while CCE is facilitated by the combination of *β* and *J*, it is usually optimized close to *J* = 1, or simple frequency-dependence, which we suggest was significant in human evolution. Third, when popularity bias increases to conformist bias *J* > 1, changes in median utility gains become punctuated, with spikes in variance before each leap that may serve as early warning signals. It is worth noting that we do not incorporate any extra (e.g. cognitive) mechanisms to account for the emergence of punctuated changes [17,89]. That is, the model shows how gradual and punctuated change are two sides of the same basic set of evolutionary rules. Conformist bias also induces homophily, where suboptimal groups lag behind the population’s best utility. When the conformist bias becomes too strong, CCE ceases totally, which might be a dramatic transition.

In terms of cultural evolution, the parameters of information transparency and popularity bias are fundamental [56]. Consistent with expectations, popularity bias and information transparency combine to maximize CCE. While the best combination depends on other parameters, generally innovation is optimized near *J* = 1. When popularity bias is increased from frequency dependence (*J* = 1) into conformist bias (*J* > 1), it begins to hinder innovation until eventually (*J* ≫ 1) collapses into a single choice. Conformist bias (*J* > 1) also underlies a punctuated mode of change. As *J* is increased beyond 1, stasis periods emerge and then become longer as *J* increases. The pauses in CCE reflect clustering of agents that become stuck at suboptimal utility values through conformist bias toward popular traits. In a process resembling homophily, these maladaptive groups emerge without any network structure [23,30] or changes in invention process [17] imposed upon the model.

In the model, increasing *J* modestly above 1 can compensate for a decrease in information transparency *β*. Conversely, the effects of excessive conformity (*J* ≫ 1) can be countered by increasing information transparency *β*, but the larger population size *N*, the more transparency is needed to overcome the utility barriers induced by conformist bias. Increasing *J* too far, without increasing *β* to compensate, stalls CCE. The relationship between CCE and population size depends on the ratio of *J* − 1 to *β*. This helps contextualize the discussion of CCE and whether improvements can be discovered in large populations [19,31,69,90,91].

Among our goals has been to establish a foundation for understanding the future of cultural evolution using fundamental principles that applied in the deep past. In terms of the latter, we find it significant that CCE is optimized near *J* = 1, equivalent to simple frequency-proportional copying. This result was unexpected, as we did not model any cost–benefit function [92] to facilitate it. As this was a robust outcome of the model, we suspect that it has wider evolutionary significance. We hypothesize that a cognitively frugal copy-popular strategy, was a natural starting point for the further evolution of the uniquely human Social Brain [93,94]. This would be similar to the copy-recent strategy that allows a majority to follow the well-informed minority [95–101], analogous to high *β* and *J* ≈ 1. As hominin groups became larger in the last two million years [102], they would have benefited from conformist bias, *J* > 1, made possible by cognitive evolution towards numeracy and working memory [103,104]. In human groups, conformity can enhance group cooperation and learning through the collective awareness of shared attention [9,47,105,106].

Subsequent modifications of this model could incorporate a parameter for memory, as reputation—memory of an individual’s interaction history [59]—affects levels of reciprocity among humans and non-human primates [48,107–109] with different functional network components of the brain activated for direct and indirect reciprocity [110–112]. Other parameters could include decay of intrinsic utility with age [113], emotions [114], boom-bust population dynamics [115], variable invention rates [17,82,116] or intrinsic preferences [81]. Parameters should be added incrementally, however, as each will multiplicatively increase the complexity of outcomes. Another question is how our results would change under skewed error distributions, such as the Gumbel distribution [22,69,90], such that utility barriers can be exceeded more often. Also, our parameters were applied to the entire simulated population; future work could model subpopulations with different *β* and *J* values.

As the model accords with other approaches to CCE featuring popularity bias as a key parameter [117,118], the additional parameter of transparency is relevant to contemporary contexts [119–121]. Popularity bias is exacerbated by algorithms that prioritize recent and popular information [119,122], such that low-cost, low-utility information is copied across massive online networks [123–125]. In the modern era, information is often not transparent, and popularity is no longer the best proxy for quality. Contemporary social media, for instance, do not necessarily surface the best ideas. Instead, online homophily, or the tendency for similar people in social groups to be connected together, has frequently polarized ideas and beliefs [126–129]. For example, clear pay-offs often lose out to the spread of misinformation [119,124,130–136]. In addition, such a large number of competing, similar options exist that informed, social learning may not be the best null hypothesis—thousands of daily social media influences amid billions of users, tens of thousands of discernible topics [125] and thousands of daily decisions [137].

For understanding online misinformation, a key factor should be how *β* and *J* act upon the sharing of information that are explicitly labelled by popularity (likes) as well as varying micro-levels of intrinsic utility, iterated over thousands/millions of online actions [61,124]. The interaction between *β* and *J* indicates a reason why culture with massive numbers of users (large *J* and *N*, low *β*) arguably change continually without necessarily getting ‘better’ [61,66,91,138–140]. Additionally, the homophily under conformity in the model resembles the well-known sorting and polarization in social media and politics [129,134,135,141].

For these reasons, the model of CCE we have presented could help explore how popularity bias and transparency of information have been pivotal from the evolution of social learning to contemporary culture. In the future, the model could be used as a foundation for connecting the evolution of the Social Brain, in small groups of transparent social learning, towards anticipating cumulative ‘cultural evolution in the digital age’ [119] and its limitless options of varying quality and degree of social conformity.

## 5. Methods

### 5.1. Emergence of utility barriers

In our model, reproduction rate of each variant *i* depends on both its utility *U*_*i*_ and trait frequency *p*_*i*_ (2.3). If there is no transparency (*β* = 0) and popularity bias is small (*J* ≈ 0), however, then their is neither population bias nor selection based on utility, and all traits are equally probable *Pr*(*U*_*i*_) ≈ 1/*k*, where *k* is the number of different variants (see equation (2.4)). If *β* = 0 and *J* ≈ 0 and also there are no new innovations introduced, *μ* ≈ 0, then eventually one variant will become fixed, i.e. the homogeneous choice, with probability 1/*k* [72].

Alternatively, assume a homogeneous population with *J* > 1, *β* > 0 and innovation rate *μ* > 0, in which all but one of the *N* agents, fraction 1 − 1/*N*, have a variant with utility *U*_0_ or less. The single agent, fraction 1/*N*, has chosen (or invented) a better variant, with utility *U*_0_ + Δ_*U*_, where Δ_*U*_ > 0 (figure 6*a*). Here, the propensity (2.5) of choosing the variant with utility *U*_0_ is

$$\Pi (U_{0},1-1/N)={\left(1-\frac{1}{N}\right)}^{J}\;e^{\beta U_{0}+\psi }\equiv \Pi_{0},$$

while the propensity of choosing the better variant is increased by a factor of $e^{\Delta_{U}}$

$$\Pi (U_{0}+\Delta_{U},1/N)={\left(\frac{1}{N}\right)}^{J}\;e^{\beta U_{0}+\beta \Delta_{U}+\psi }\equiv \Pi_{\Delta }.$$

We hypothesize that there is a critical utility improvement, $\Delta_{U}>\Delta_{U_{c}}$, such that we expect the variant to be chosen at least once in the next interval, among the *N* selections of the *N* agents. As a mean-field approximation, this occurs when the variant can be expected to be selected by at least one agent (a conservative assumption, as this allows for it to be selected multiple times as well)

$$N\left(\frac{\Pi_{\Delta }}{Y_{t}}\right)\geq 1,$$

where the normalization term equals the sum of the propensities, i.e. $Y_{t}=\Pi_{0}+\Pi_{\Delta }$. This condition may be rewritten as

$$\Pi_{\Delta }(N-1)\geq \Pi_{0}.$$

By taking natural logarithms of both sides of the previous equation, we obtain

$$\ln(N-1)+J\ln\left(\frac{1}{N}\right)+\beta \Delta_{U}+\beta U_{0}\geq J\ln\left(1-\frac{1}{N}\right)+\beta U_{0}.$$

Hence, the critical utility barrier ($\Delta_{U_{c}}$) relates to the model parameters as follows:

$$\Delta_{U}\geq \Delta_{U_{c}}=\frac{J-1}{\beta }\ln(N-1).$$

This inequality predicts the critical utility barrier grows with the logarithm of population size (*N*) and popularity bias (*J*). Note we have assumed all alternative variants have utility *U*_0_ *or less*, so this is a conservative threshold.

Figure 6*c* compares the theoretical estimate for $\Delta_{U_{c}}$ (blue line) with numerical simulations for various parameter values and multiple choices. The simulations show our theoretical prediction applies even to a non-homogeneous population. Multiple new variants could exceed the utility barrier; due to stochastic factors, it is impossible to anticipate which one of these inventions will finally be transmitted; all that can be predicted is that at least one of those located beyond the critical utility barrier will survive to the next generation.

### 5.2. The pace of cultural evolution

The pace of CCE is driven by the rate of invention *μ* and social adoption (the interaction between popularity bias *J* and transparency *β*). Stochastic effects are important here because populations would become stationary and incapable of adapting to environmental changes if there is no source of variation. In this context, populations can improve existing utilities (*U*_*t*_) by performing many small random changes to them, i.e. *U*_*t*_ + Δ → *U*_*t*+1_. What is the probability *P*(*U*_*t*+1_ > *U*_*t*_) of making this transition? How probable it is that innovations drive populations towards greater utility?

Let us assume that random changes (Δ) follow a normalized Gaussian distribution N(Δ). Given the symmetry in the normal distribution, changes can be either beneficial (Δ_+_) or deleterious (Δ_−_) (figure 5*a*). That is, we will observe utility increases when there are beneficial but not deleterious change events

$$P(U_{t+1}>U_{t})=\;P(\Delta_{+}\cap \neg \Delta_{-})=P(\Delta_{+})-P(\Delta_{+}\cap \Delta_{-}),$$

where the first term on the right side defines the probability of beneficial change

$$P(\Delta_{+})=\int_{0}^{\infty }N(\Delta )\;d\Delta =\frac{1}{2}.$$

and the second term is the probability of maladaptative and neutral changes. As previously stated, variations of size Δ must overcome the utility barrier to be inherited in the next time step, i.e. $\Delta >|\Delta_{U_{c}}|$. Taking into account the utility barrier, this probability can be expressed as follows:

$$P(\Delta_{+}\cap \Delta_{-})=\int_{0}^{\left|\Delta_{U_{c}}\right|}N(\Delta )\;d\Delta .$$

When we put these definitions into the first equation, we get the following expression for the probability of increasing population utility:

5.1 $$P(U_{t+1}>U_{t})=\frac{1}{2}-\int_{0}^{\left|{\Delta_{U}}_{c}\right|}N(\Delta )\;d\Delta .$$

This expression implies that the maximum rate of improvement (1/2) is restored at *J* = 1, i.e. when there is no utility barrier ($\Delta_{U_{c}}=0$) and maladaptive and neutral changes are excluded.

### 5.3. Approaching the optimal popularity bias

The popularity bias *J* > 1, which amplifies the pace of cultural evolution as a function of degree of transparency *β* and population size *N*, is approximated in this section. According to computer simulations, removing maladaptive and neutral changes enhances the rate of improvement dramatically. Theoretical arguments suggests this takes place in the vicinity of the *J* = 1 transition (see above section and the peak in figure 3*b*). On the other hand, evolutionary stasis is prolonged when popularity bias is too strong, only amplifying the current dominant trait. What is the optimal value of *J* = *J*_*s*_ that discards irrelevant changes without slowing down evolution?

We can use a geometrical argument to estimate the popularity bias’s value. The utility barrier Δ_*U*__*c*_(*J*_*s*_) should be as near to the Gaussian distribution’s centre as allowed, so that most neutral changes (however minor) are ignored (figure 5*a*). Formally, we need to solve the following equation:

$$\int_{{\Delta_{U}}_{c}(J_{s})}^{\infty }N(\Delta )\;d\Delta =\frac{1}{2}\int_{0}^{\infty }N(\Delta )\;d\Delta ,$$

where the left- and right-hand sides represent the probability of adaptive changes (5.1) and the probability of discarded positive changes, respectively. Using the exponential approximation for the complementary error function

$$\int_{{\Delta_{U}}_{c}(J_{s})}^{\infty }N(\Delta )\;d\Delta =\frac{e^{-{\left({\Delta_{U}}_{c}(J_{s})/2\right)}^{2}}}{2},$$

we can obtain the equivalent expression

$$e^{-{\left({\Delta_{U}}_{c}(J_{s})/2\right)}^{2}}=\frac{1}{2}.$$

At this point, we can substitute the definition of the utility barrier Δ_*U*__*c*_(*J*_*s*_) (following equation (3.1)) to find that popularity bias *J*_*s*_ satisfies this condition

$$\frac{J_{s}-1}{\beta }\log(N-1)=\pm 2\sqrt{\log(2)}.$$

Taking the positive boundary,

$$J_{s}=1+\frac{\beta }{\log(N-1)}2\sqrt{\log(2)}.$$

This equation suggests that the value of the popularity bias *J*_*s*_ optimizing the pace of cultural evolution is a balance between transparency and population size. Increasing the transparency of information *β* > 0 pushes the popularity bias *J*_*s*_ away from 1 (and increasing the likelihood of neutral changes). On the other hand, popularity bias *J*_*s*_ ≈ 1 for large population sizes (*N* → ∞).

### 5.4. Effect of population size

In this work, we hypothesized that utility barriers occur when there is a large popularity bias *J* > 1. The rate of cultural change is determined by the location of the utility barrier, which is influenced by both transparency and population size (see previous section). The likelihood of evolutionary slowdown increases as the utility barrier rises. It is also possible that the effect of frequency-dependence copying is so powerful for large population sizes that it halts the evolutionary process.

To compute the size-dependent bounds of popularity bias *J*_*C*_ > *J*, we estimate the probability that no one in the population finds a variant crossing the utility barrier Δ_*U*__*c*_(*J*_*C*_). As before, the probability of adaptive changes is

$$\int_{{\Delta_{U}}_{c}(J_{C})}^{\infty }N(\Delta )\;d\Delta =\frac{e^{-{\left({\Delta_{U}}_{c}(J_{C})/2\right)}^{2}}}{2}.$$

Using a mean-field approach, we assume that the maximum popularity bias corresponds to an average number of individuals discovering an innovation being less than one

$$\frac{N}{2}\exp\left[-{\left(\frac{J_{C}-1}{2\beta }\log(N-1)\right)}^{2}\right]<1.$$

After some algebra, we arrive at the expression

$$\log\left(\frac{2}{N}\right)>\frac{{\left(J_{C}-1\right)}^{2}}{2\beta^{2}}\log(N-1).$$

At this point, we can extract a limit of popularity bias *J*_*C*_ > *J* that depends on the population size *N*

$$J_{C}=1+\beta \sqrt{\frac{2\log(N/2)}{\log(N-1)}}.$$

For large population *N* with *J* > 1, we obtain the following precondition for CCE:

$$J_{C}(N\to \infty )=\lim_{N\to \infty }1+\beta \sqrt{\frac{2\log(N/2)}{\log(N-1)}}∼1+\beta \sqrt{2},$$

which can be alternatively described as

5.2 $$\frac{J-1}{\beta }\leq \sqrt{2}.$$

This equation suggests that in order to observe cumulative cultural behaviour, the ratio between conformity and transparency must be bounded by the corrected variance of the innovation distribution. Above this threshold, the probability of reaching higher utility values decreases exponentially.

## Data Availability

The data are provided in electronic supplementary material [142].
